# Fully Arthroscopic Latissimus Dorsi Transfer for Irreparable Posterosuperior Massive Rotator Cuff Tears

**DOI:** 10.1016/j.eats.2025.103447

**Published:** 2025-02-03

**Authors:** Lin Lin, Hui Yan

**Affiliations:** Department of Sports Medicine, Peking University Third Hospital, Institute of Sports Medicine of Peking University, Beijing, China

## Abstract

Latissimus dorsi (LD) transfer can be used for the treatment of irreparable posterosuperior massive rotator cuff tears. This article describes a fully arthroscopic LDT procedure to manage irreparable supraspinatus or infraspinatus tears. Dissection and release of the whole LD tendon and muscle are performed arthroscopically. Great attention must be paid to avoid the neurovascular structures during the procedure. Using a portal superior to the upper margin of the pectoralis major (PM) and a portal inferior to the lower margin of the PM, partial detachment of the proximal PM tendon is not necessary to obtain access to the insertion of the LD tendon. By enlarging the posterior axillary portal to 2 to 3 cm in length, the LD tendon can be easily pulled out of the skin and is weaved in an open way. The muscular part of the LD can be further released to a sufficient length, and the posterior pathway of the LD transfer is created arthroscopically. The LD tendon can be transferred posteriorly to the greater tuberosity of the humeral head and fixed with 2 knotless anchors.

Management of irreparable massive rotator cuff tears remains challenging.^1^ Tendon transfers have been used for irreparable posterosuperior (PS) massive rotator cuff tears.^2^^,^^3^ Posterior latissimus dorsi (LD) transfer (LDT) has shown satisfactory outcomes for recovery of shoulder function at long-term follow-up regardless of patient age.^4^, ^5^, ^6^

Traditionally, LDT procedures were performed in an open way for PS transfer.^5^ Techniques for arthroscopy-assisted and fully arthroscopic LDT have been described with the advantages of better visualization of the neighboring neurovascular structures at risk and no damage to the deltoid muscle.^7^, ^8^, ^9^, ^10^, ^11^, ^12^, ^13^, ^14^, ^15^ We describe a modified arthroscopic technique with some advantages in tendon harvesting and releasing. This procedure could be used for PS transfer of the LD.

## Surgical Technique

The operation is performed with the patient under general anesthesia with an interscalene regional block. The operative technique is shown in Video 1, and pearls and pitfalls of the procedure are presented in Table 1.

**Table 1:** Pearls and Pitfalls of Fully Arthroscopic LD Transfer

Step	Pearls	Pitfalls
1	Release of retracted rotator cuff to facilitate partial repair	Irreparable cuff tear
2	Identification of axillary nerve crossing through quadrilateral space and dissection medial to LHT to avoid injury to axillary nerve Identification of teres major and LD muscle Release to enlarge pathway of transferred tendon Release of connections between skin and LD muscle to ufficient length	Injury to axillary nerve Over-swelling of posterior soft tissue Narrow pathway for posterior transfer Shortening of LD excursion
3	Use of LHBT, subscapularis tendon, 3 sisters, conjoint tendon, and superior margin as landmarks to identify LD tendon Identification of axillary nerve, as well as radial nerve and its branches to LHT Dissection of upper and lower borders of LD, with release as far as possible and cutting of aponeurotic band Detachment of LD tendon just medial to LHBT	Chronic LHBT rupture and significant scar tissue around conjoint tendon and subscapularis tendon Axillary and radial nerve injury Shortening of LD excursion Shortening of LD tendon and injury to teres major tendon
4	Pulling out from posterior axillary portal Preparation of LD tendon with different-color sutures to avoid twisting of tendon when transferring LD Further release of LD muscle and evaluation of LD excursion	Non-thorough release of LD Twisting of tendon when passing tendon to subacromial space LD pedicle injury
5	Use of correct pathway created in step 2 for posterior transfer Fixation with 2 knotless anchors and third fixation point of transferred tendon	Axillary nerve compression Poor LD healing and poor bone quality

LD, latissimus dorsi; LHBT, long head of biceps tendon; LHT, long head of triceps.

### Patient Positioning and Portal Placement

**Fig 1:**
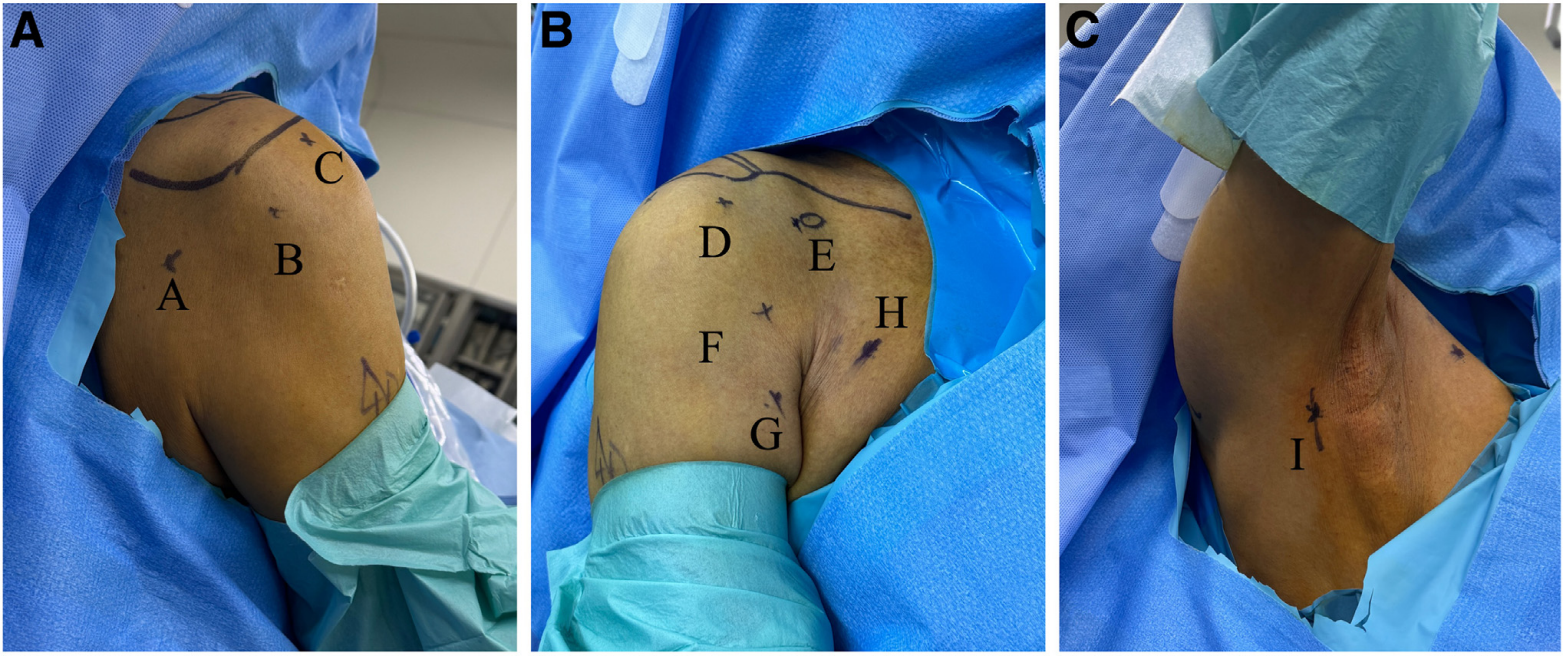
(A-C) Portals used for fully arthroscopic latissimus dorsi (LD) transfer in right shoulder in beach-chair position: lower posterior portal (portal A), located 1 cm medial and 3 to 4 cm inferior to posterolateral corner of acromion; posterolateral portal (portal B), lateral portal (portal C); anterolateral portal (portal D), located just 3 cm anterior and lateral to anterolateral corner of acromion; anterior portal (portal E), lateral to coracoid tip; portal superior to upper margin of pectoralis major (portal F), used for visualization and LD release; portal inferior to lower margin of pectoralis major (portal G), used for LD tendon release; anterior axillary portal (portal H), located 1 finger-width medial to axillary fold and 3 to 4 finger-widths distal to coracoid tip and used for visualization and release of LD; and posterior axillary portal (portal I), used for release of LD muscle.

The patient is placed in the beach-chair position. The portals are shown in Figure 1: (1) lower posterior portal, located 1 cm medial and 3 to 4 cm inferior to the posterolateral corner of the acromion; (2) posterolateral portal; (3) lateral portal; (4) anterolateral portal, located just 3 cm anterior and lateral to the anterolateral corner of the acromion; (5) anterior portal, located lateral to the coracoid tip; (6) portal superior to the superior margin of the pectoralis major (PM) (sPM), used for visualization and LD release; (7) portal inferior to the inferior margin of the PM (iPM), used for LD tendon release; (8) anterior axillary portal, located 1 finger-width medial to the axillary fold and 3 to 4 finger-widths distal to the coracoid tip and used for visualization and release of the LD; and (9) posterior axillary (PA) portal, used for release of the LD muscle.

### Step 1: Arthroscopic Evaluation and Treatment of Concomitant Shoulder Pathologies

Lesions of the labrum, cartilage, and long head of the biceps tendon (LHBT) are evaluated and treated. After confirmation of the irreparability of the supraspinatus or infraspinatus, PS LDT is indicated.

### Step 2: Creation of Posterior Pathway of PS LDT

**Fig 2:**
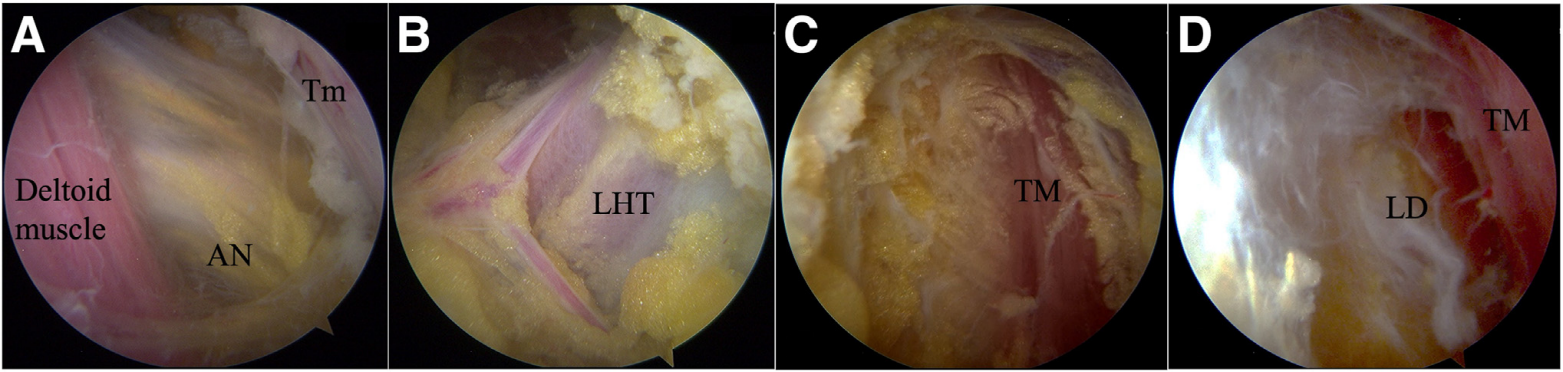
Essential landmarks for creation of posterior pathway of posterior latissimus dorsi (LD) transfer in right shoulder in beach-chair position. (A) Axillary nerve (AN) crossing through quadrilateral space between distal deltoid aponeurosis and teres minor (Tm) with arthroscope in lateral portal. (B) Identification of vertical fibers of long head of triceps (LHT) with arthroscope in lateral portal. (C) Visualization of superior margin of teres major (TM) muscle with arthroscope in posterolateral portal. (D) Identification of LD muscle and release of connections between subcutaneous fascia and surface of LD muscle with arthroscope in posterolateral portal.

Acromioplasty is not performed to prevent possible superior escape of the humeral head. Dissection is performed posteriorly using an electrocautery device (Arthrocare; Smith & Nephew, Andover, MA). First, the scope is in the lateral portal and the electrocautery device is in the posterolateral portal. Dissection is started inferiorly and posteriorly to find the axillary nerve crossing through the quadrilateral space between the distal deltoid aponeurosis and the teres minor (Fig 2A). Second, dissection is continued medially and inferiorly to identify the vertical fibers of the long head of the triceps (LHT) (Fig 2B). Furthermore, release is continued inferiorly and medially until the superior margin of the TM muscle is visualized (Fig 2C). Finally, the scope is placed in the posterolateral portal and the electrocautery device is introduced from the lower posterior portal. Release is performed along the surface of the TM to the LD muscle (Fig 2D). Typically, the space between the TM muscle and LD muscle is obvious and easy to identify. The PA portal is established with a needle and used to further release the connections between the subcutaneous fascia and the surface of the LD muscle as possible. The connections of skin and infraspinatus and/or teres minor fascia should be widely released to avoid limiting the course of the LD tendon and muscle when PS transfer is performed.

### Step 3: Anterior Release and Dissection of LD Tendon

**Fig 3:**
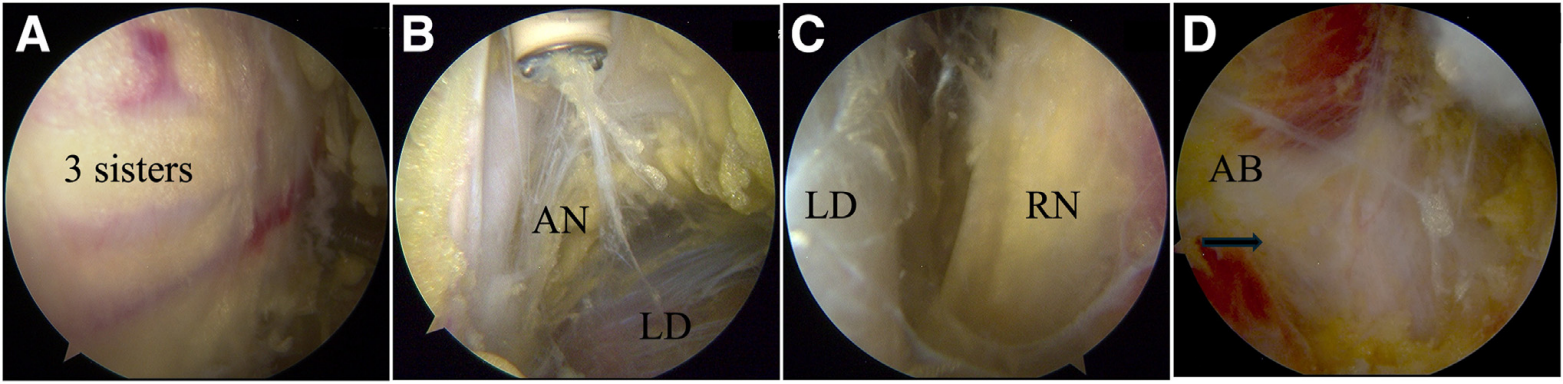
Latissimus dorsi (LD) tendon release and dissection in right shoulder in beach-chair position with arthroscope in superior pectoralis major portal. (A) Three sisters (terminal branches of circumflex vessels). (B) Axillary nerve (AN) and posterior humeral circumflex vessels superior to upper border of LD. (C) Radial nerve (RN) and its motor branch anterior to LD. (D) Aponeurotic band (AB) between inferior border of LD and triceps (balck arrow: aponeurotic band).

The scope is placed in the lateral portal, following the LHBT to reach lateral to the conjoint tendon (CT) and the upper border of the PM. At the inferior border of the subscapularis tendon, the 3 sisters (i.e., the terminal branches of the circumflex vessels) are identified, marking the upper border of the LD tendon insertion (posterior to the PM insertion) (Fig 3A).

A needle is used to position the sPM portal, which is just superior to the upper border of the PM and medial to the LHBT. Through the sPM portal, the LD tendon posterior to the PM and CT can be seen. The iPM portal is just inferior to the lower border of the PM and can be used to release the LD tendon and muscle. A switching stick can be used to elevate the CT through the anterior axillary and anterior portals.

It is not necessary to release the PM insertion with the scope in the sPM portal. The LD is thus exposed inferiorly and medially. Before the insertion of the LD tendon is detached, its upper and lower borders should be identified and released from the TM muscle. Great care is taken to prevent any injury to the axillary nerve, the circumflex vessels (quadrilateral space) (Fig 3B), and the radial nerve and its branch to the LHT (Fig 3C). The aponeurotic band between the inferior border of the LD and the LHT must be released; otherwise, it will limit the mobility of the transferred LD (Fig 3D). Follow the tendon and muscle of the LD, release should be performed as far as the limitation of the scope visualization.

**Fig 4:**
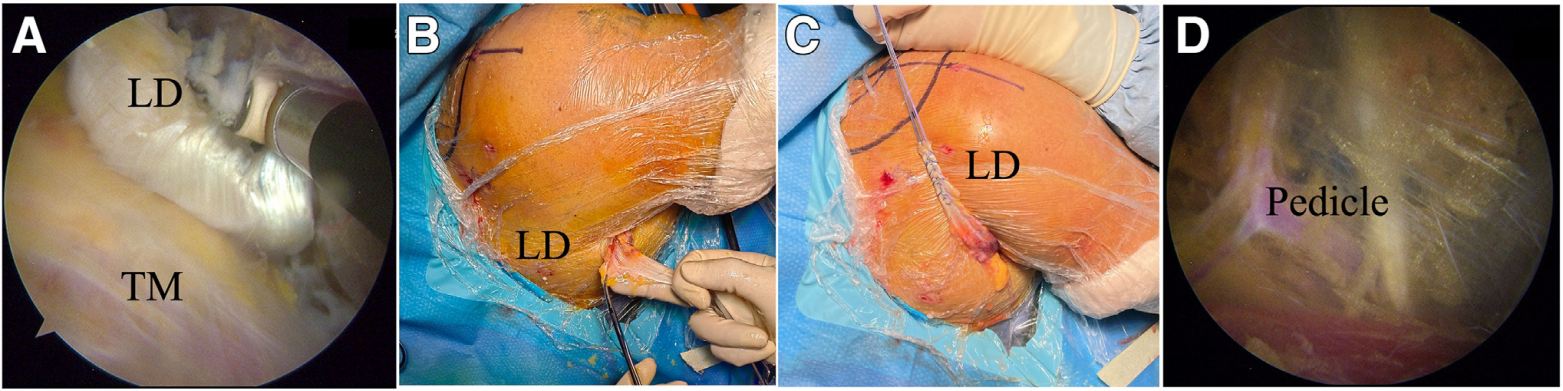
Latissimus dorsi (LD) tendon preparation and further release in right shoulder in beach-chair position. (A) Detachment of LD insertion with arthroscope in superior pectoralis major portal and cautery device in inferior pectoralis major portal. (B) The LD tendon is pulled out of the posterolateral portal and is prepared with sutures (No. 2 Orthocord sutures). (C) Neurovascular pedicle of LD muscle. (TM, teres major.)

Then, the insertion of the LD should be released from the humeral shaft to achieve the maximal length while preserving the insertion of the TM (Fig 4A). The release of the LD is continued as far medially as possible with caution to protect the radial nerve and its branch. Care must taken to separate the LD and TM muscles because their muscles are adherent on their dorsomedial edges. The connections between the LD and skin can be released all the way to the PA portal.

### Step 4: Posterior LD Tendon Preparation and Further Release

The LD tendon can be easily taken out of the skin if a sufficient release is performed arthroscopically after the PA portal is enlarged to 2 to 3 cm in length (Fig 4B). The tendon should be weaved with strong sutures (No. 2 Orthocord sutures; DePuy Mitek, Raynham, MA) at 4 to 5 cm in length (Fig 4C). This process can be performed arthroscopically. However, it is more easily performed and stronger suture can be obtained in an open way. Through this portal, the LD and TM muscle bellies can be further released. With the scope placed in the PA portal, the muscle belly of the LD from the surrounding structures, including the TM muscle, scapula, triceps, and pedicle, can be released. The posterior and superficial border of the LD can be safely released from the skin, whereas the anterior release of the LD must be performed cautiously because of the pedicle entering the muscle. The pedicle can be identified arthroscopically, and the fat tissue around it should be conserved (Fig 4D).

### Step 5: Posterior Transfer and Fixation of LD

**Fig 5:**
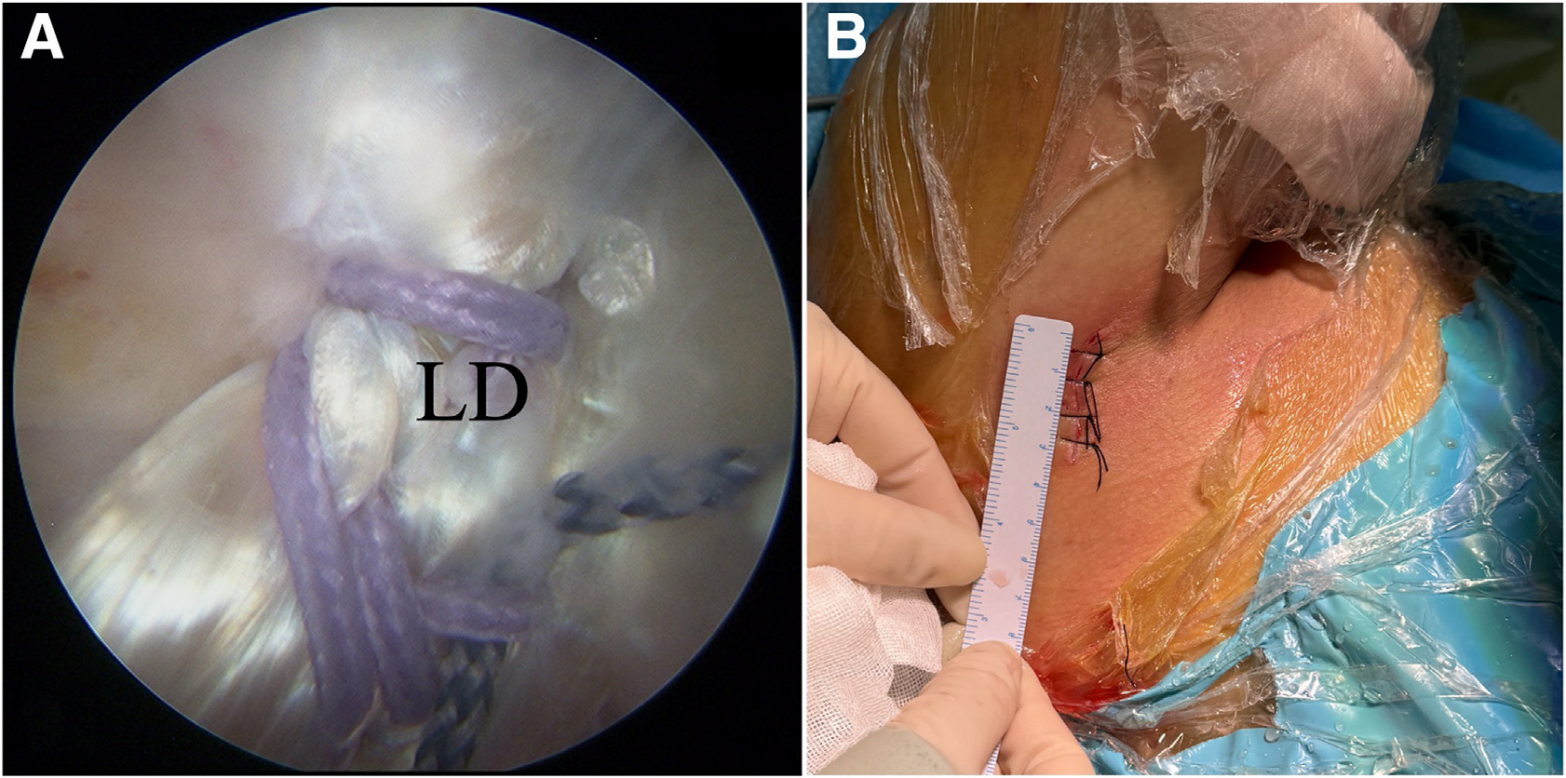
Latissimus dorsi (LD) tendon transfer and fixation. (A) Posterior transfer of LD tendon in right shoulder in beach-chair position and fixation of LD tendon with two 4.75-mm knotless anchors (SwiveLock) on greater tuberosity with arthroscope in lateral portal. (B) Incision of 3 cm in length.

A grasper with PDS II suture (Ethicon, Somerville, NJ) is placed in the posterolateral portal and enters the posterior pathway to shuttle the LD tendon with the scope in the lateral portal. The sutures might be retrieved through 2 different portals to avoid the risk of mistakes between the 2 loaded sutures. Lateral sutures should be fixed on the lateral of the greater tuberosity and medial sutures should be fixed on the medial of the greater tubeosity. After partial rotator cuff repair with suture anchors (4.5-mm Corkscrew; Arthrex, Naples, FL) if possible, the LD tendon can be fixed with two 4.75-mm knotless anchors (4.75-mm SwiveLock; Arthrex) on the greater tuberosity. The sutures on the anchors for partial rotator cuff repair can be used as the third fixation site for the transferred LD tendon (Fig 5A). Figure 5B shows that the incision for the PA portal measures 3 cm.

### Postoperative Rehabilitation

The patient is immobilized with an abduction pillow for 6 to 8 weeks. Passive shoulder range-of-motion exercise is started at 2 weeks postoperatively. After 6 weeks, the patient begins slow active rehabilitation. After 3 months, slow strengthening exercises are started.

## Discussion

Many different techniques for LD tendon transfer have been described, including open surgery and arthroscopy-assisted, mini-open, and all-arthroscopic techniques.^9^, ^10^, ^11^, ^12^^,^^14^^,^^16^ In our technique, dissection and release of the whole LD tendon and muscle are performed arthroscopically. Partial detachment of the proximal insertion of the PM tendon is not necessary to obtain access to the insertion of the insertion of the LD tendon using the sPM and iPM portals. By enlarging the PA portal, the LD tendon can be easily retrieved out of the skin and stronger suture can be obtained in an open way compared with preparation arthroscopically.

The present technique is modified based on previous techniques described by Kany et al.^15^ and Lopez-Fernandez et al.^12^ A combined fully arthroscopic technique for transfer of the LD and TM has been proposed in which the tendons are fixed at the junction of the supraspinatus and infraspinatus to decrease the failure rate.^14^ According to our experience, the TM tendon can be detached and released in the same way using the present technique—and can be transferred posteriorly and fixed in a lower position compared with fixation of the LD. Because the course of the TM tendon and muscle unit is shorter compared with LD tendon and muscle unit and would decrease the excursion of the transfer when fixation with LD together.

The main limitation of this procedure is the steep learning curve. There is always a risk of injury to the neurovascular structures. The present technique requires improved arthroscopic skills with expert knowledge of the anatomy. Cadaveric training is helpful and recommended.

## Disclosures

Both authors (L.L., H.Y.) declare that they have no known competing financial interests or personal relationships that could have appeared to influence the work reported in this paper.

## Supplementary Data

Video 1 Fully arthroscopic latissimus dorsi transfer for irreparable posterosuperior massive rotator cuff tears. The patient is placed in the beach-chair position. The superior pectoralis major portal, inferior pectoralis major portal, anterior axillary portal, and posterior axillary portal are designed to release the latissimus dorsi tendon. After arthroscopic examination, the massive rotator cuff tear is confirmed to be irreparable; latissimus dorsi transfer is indicated. Dissection is performed posteriorly using an electrocautery device. The axillary nerve is identified. Dissection is continued medially and inferiorly to identify the vertical fibers of the long head of the triceps. Release is continued until the superior margin of the teres major muscle is visualized. The scope is placed in the posterolateral portal, and the electrocautery device is introduced from the lower posterior portal. Release is performed along the surface of the teres major to the latissimus dorsi muscle. Then, the scope is placed in the anterolateral portal. The 3 sisters are identified. The upper border of the pectoralis major is exposed. Then, the superior pectoralis major portal is established. The anterior axillary portal is also established. A switching stick is used to expose the latissimus dorsi. The scope is placed in the superior pectoralis major portal, and the upper border and lower border of the latissimus dorsi are exposed. Great care is taken to prevent any injury to the axillary nerve and radial nerve. The connections between the latissimus dorsi and skin can be released. The aponeurotic band between the inferior border of the tendon and the long head of the triceps must be released. The insertion of the latissimus dorsi is released from the humeral shaft. Care must taken to separate the latissimus dorsi and teres major muscles because their muscles are adherent on their dorsomedial edges. The release should be performed until the posterior axillary portal is reached. The pedicles to the latissimus dorsi can be seen. The posterior axillary portal is enlarged to 2 to 3 cm, and the latissimus dorsi tendon can be easily retrieved out of the skin. The tendon is weaved with Orthocord sutures. A grasper with PDS II suture is used to bring back the latissimus dorsi tendon through the posterior pathway. The tendon and sutures are fixed to bone with 2 knotless anchors. The incision measures less than 3 cm.
